# No strings attached: a mixed methods evaluation of the acceptability of the San Francisco Abundant Birth Project, a perinatal guaranteed income program

**DOI:** 10.1186/s12889-026-26300-z

**Published:** 2026-01-22

**Authors:** Monica M. De La Cruz, Stephanie Arteaga, Erin M. Hubbard, Reiley D. Reed, Wyconda Cotton-Curtis, Michaela Taylor, Breezy Powell, Anjeanette Coats, Sabra Bell, Payshia Edwards, Maile Chand, Troy Roberts, Brittany Chambers Butcher, Deborah Karasek, Zea Malawa, Anu Manchikanti Gomez

**Affiliations:** 1 Sexual Health and Reproductive Equity Program, School of Social Welfare, University of California, Berkeley, USA; 2 Expecting Justice, Oakland, CA USA; 3 University of California, Davis, USA; 4 Oregon Health and Sciences University, Portland, USA

**Keywords:** Guaranteed income, Pregnancy, Community-centered, Birth equity

## Abstract

**Background:**

Economic insecurity during pregnancy is a critical social determinant of maternal and infant health. Traditional public assistance programs often fail to meet the needs of marginalized communities, particularly Black and Pacific Islander (Pasifika) pregnant women and people. Guaranteed income (GI)–unconditional cash transfers for specific populations–has emerged as a potential solution, but little is known about the experiences of participants accessing perinatal GI programs. The current study describes the implementation and acceptability of the San Francisco Abundant Birth Project (ABP), the first perinatal GI pilot program in the U.S. ABP provided $1,000 per month for up to 14 months to 151 Black and Pasifika pregnant individuals in San Francisco, CA, between 2021 and 2024.

**Methods:**

We conducted a mixed-methods participatory evaluation with a subset of program participants (116 survey and 21 interview participants), and asked about program satisfaction, trust, and experience. Community researchers co-led the study design, data collection, and analysis.

**Results:**

Participants reported being very satisfied (95.7%) with ABP and felt very respected by the staff (95.7%). A majority (77.5%) trusted ABP to act in their best interest, compared to 9.0% who trusted the government to do the same. While some participants initially doubted that the funds from ABP were unconditional, trust grew over time. Qualitative findings emphasized the importance of ABP’s community-centered design and culturally aligned staff in building trust.

**Conclusion:**

Findings highlight the value of community-rooted GI programs in fostering trust, dignity, and reimagined models of pregnancy support.

## Background

Economic insecurity during pregnancy is a critical social determinant of maternal and infant health. Chronic stress from economic insecurity, including lack of secure housing, food, stable prenatal care and exposure to discrimination, heightens the risk of maternal anxiety, depression, and postpartum mood disorders, which can affect infant health and parent-child bonding [1–4]. Lower income during pregnancy is associated with higher and more sustained levels of maternal cortisol, a stress hormone [5, 6]. Maternal cortisol can cross the placenta, with elevated levels potentially disrupting infant development, leading to preterm birth, low birth weight, and structural changes in brain regions associated with cognition and emotional regulation [7, 8]. For minoritized populations, the impacts of stress can be further exacerbated by systemic inequities such as structural racism and inadequate access to culturally competent care [8, 9].

Although social safety net programs like Temporary Assistance for Needy Families (TANF) exist in the United States (U.S.), they frequently fail to provide economic security for pregnant individuals, especially those from marginalized communities facing intersecting economic and health disparities [10–12]. TANF’s restrictive eligibility criteria, coupled with a system often marked by surveillance and administrative burden, frequently undermine the security needed to support long-term maternal and infant health and well-being [11, 13–15].

There is an urgent need for innovative, community-centered interventions aimed at addressing family economic insecurity, and guaranteed income (GI) programs have emerged as a promising solution. GI programs provide people with regular, unconditional cash transfers, meaning that recipients do not need to do anything to receive the money, and they can spend it however they see fit [16]. There are over 160 GI pilot programs that have been or are currently being tested across the U.S. to assess whether GI results in positive outcomes among participants with the goal of creating sustainable policies [17]. These pilots vary in their populations of focus (e.g., young people, recently incarcerated) and are typically limited to specific geographic areas.

The San Francisco Abundant Birth Project (ABP) was the first pregnancy GI pilot program in the U.S, providing cash in the prenatal and postpartum periods. Specifically, the ABP pilot aimed to positively impact birth outcomes through increasing economic security among two groups in San Francisco with the highest rates of preterm birth, the Black and Pacific Islander (Pasifika) communities. ABP was the signature initiative of the birth justice collaborative Expecting Justice and supported by a multi-sector, citywide collaboration. ABP was intentionally developed with and for Black and Pasifika community members in San Francisco, with their perspectives incorporated throughout the design, implementation, and evaluation of the program [18]. Central to the community co-creation process was the Community Researcher model, where four Black and Pasifika women with lived experience as birth workers and mothers in San Francisco were trained in research methods to participate in all aspects of the evaluation, including conducting formative research to inform the program design, planning the evaluation research design, collecting and analyzing data, and participating in dissemination activities [18]. The ABP pilot launched on Juneteenth (June 19), 2021 and ended in January 2024. To be eligible for the program, individuals had to identify as Black and/or Pasifika, live in San Francisco or be recently displaced (e.g., had moved out of San Francisco within the last 6 months), be less than 27 weeks pregnant, and have an annual household income of less than $100,000 USD. As part of the application process, applicants self-reported their income and racial identity, provided proof of residence, and submitted a letter from a healthcare provider confirming pregnancy. Individuals meeting eligibility criteria entered the Abundance Drawing, a bimonthly randomized selection process. Applicants remained in the Abundance Drawing pool until they were selected or entered the 3rd trimester and became ineligible.

The program distributed $1,000 per month during pregnancy and for 6 months postpartum, provided through a debit card that could be used for both cash withdrawals and debit card purchases. Total payments made to participants over the course of the program ranged from a minimum of $9,000 to a maximum of $14,000, with an average of $10,000 disbursed per participant; amounts varied based on when participants enrolled (i.e., those who enrolled earlier in their pregnancies received more months of income supplementation during pregnancy). After giving birth, program participants received the monthly GI supplement for a minimum of 5 months and a maximum of 7 months, with an average of approximately 6 months of payments in the postpartum period.

Once selected for the program, participants attended an orientation with program staff called Abundance Coaches, who were also birth workers from the communities the program served, and liaised with them before and during the program. The Abundance Coaches onboarded ABP participants and provided ongoing support throughout the program through regular check-ins and optional, light, individualized case management called Abundance Coaching to link to wrap-around services (e.g., housing support, food resources, financial management). The 15-minute check-ins were designed as quick opportunities for participants to connect with the coaches, and three were scheduled throughout the program: two weeks after enrollment, during their third trimester, and one month after birth. These check-ins ensured even those who had not opted into Abundance Coaching had regular touchpoints with the program; attendance was encouraged but not required and did not impact receipt of the income supplement.

The current study uses survey and interview data to describe the demographics of program participants and explore the acceptability of the San Francisco ABP pilot program. Program acceptability moves beyond participant satisfaction and is a multi-faceted construct that refers to how appropriate, reasonable, and worthwhile a participant believes a program to be, given its design [19]. We describe participants’ experiences with (1) program application and enrollment, (2) Abundance Coaching, including interactions with Abundance Coaches, (3) trust in ABP, and (4) completing the program.

## Methods

In keeping with the philosophy of GI as an unconditional, no-strings-attached intervention, individuals selected for ABP could opt in to participating in the mixed-methods evaluation. The evaluation was designed using a community-centered approach, with community researchers co-designing the survey instruments and interview guides, collecting data, and participating in data analysis and dissemination activities.

### Data sources

#### Surveys

The evaluation included four interviewer-administered surveys (administered via phone or videoconference by a community researcher or staff member) at study enrollment (anytime before the 28th week of pregnancy; T1); during the third trimester (28th week of pregnancy to birth) and at least 4 weeks after T1 (T2); 6–16 weeks postpartum (T3); and 6–7 months postpartum (T4). All participants who completed T1 were eligible for the T2, T3, and T4 surveys, even if they had missed a prior survey. Surveys took approximately 60–90 min to complete, and participants received a $100 gift card for each survey completed. Research participants provided informed consent for all surveys at the time of study enrollment. Measures used in this analysis were derived from demographic and social service participation data at T1 and program experience questions at T1-T4. The program acceptability questions were tailored to the participant trajectory, with surveys covering experiences with applying to and enrolling in the program (T1 and T2), Abundance Coaching and overall program participation (T2-T4), feelings regarding the end of the program (T4), and overall satisfaction with and trust in the program (T1-T4).

#### Program data

The ABP program shared aggregate data with the evaluation team on the number of individuals who applied, were deemed eligible, and enrolled in the program. In addition, for those that consented to research participation, individual-level program data were linked to research data. Linked program data included dates of enrollment, receipt of ABP debit card, and first and final payments.

#### In-depth interviews

We invited a subset of research participants to complete two in-depth qualitative interviews, during the third trimester of pregnancy and six months postpartum. We used purposive sampling and prioritized participants who identified as Pasifika and/or non-binary, genderqueer, or transgender, as well as those who experienced a preterm birth, to participate in qualitative interviews, given the smaller proportion of these individuals in the overall study. Among the remaining Black-identified participants, we prioritized recruiting those who: (1) did not report using public benefits, (2) were experiencing housing or food instability, or (3) expressed confidence in their ability to meet long-term financial goals while using public benefits. We included the latter to use a strengths-based approach, focusing on the positive aspects of these participants’ conceptualization of their financial health (i.e., feeling on track to meet their long-term financial goals) despite experiencing financial hardship (i.e., use of public benefits to support their income). All three groups were prioritized for qualitative study recruitment by the entire study team due to existing literature showing that: (1) higher income does not necessarily protect Black women and birthing people from adverse pregnancy and birth outcomes; (2) stressors, including housing and food insecurity, are important indicators of birth complications; and (3) research involving Black women often uses a deficits-based framework. Our approach aimed to capture a range of experiences, including those of resilience, agency, and future orientation.

Study participants who expressed interest in the qualitative study signed a separate consent form after meeting with a research staff member. Interviews were conducted remotely via video conference or phone. Two interview guides were developed with community researchers for this study and explored participants’ financial and employment stability, mental health and wellbeing, and program satisfaction both prior to and after giving birth. On average, prenatal interviews lasted 71 min, while postpartum interviews lasted an average of 74 min. Participants received a $100 gift card for each interview they completed. Interviews were audio recorded and professionally transcribed.

### Quantitative analytic approach

We first described the demographics of the ABP participant sample. We then generated descriptive statistics, including response frequencies, to summarize responses to survey questions. We analyzed program data on payment initiation and timing each month to determine the number of payments each participant received. All analyses were performed using Stata 17 statistical analysis software.

### Qualitative analytic approach

We employed a Rapid Assessment Process (RAP) to analyze the interview data [20]. RAP is a rigorous, team-based approach that includes iterative analysis and data triangulation to develop preliminary findings. Notably, this analytic process is well-suited for studies evaluating program implementation, given its ability to produce actionable findings to inform program design. First, author RDR completed a transcript summary for each interview, using a template with domains regarding program acceptability and feasibility, developed by author SA. Next, information from the transcript summaries was entered into a data matrix, organized by domain. Lastly, authors RDR and WCC developed summaries of each domain with key takeaways. SA then extensively reviewed domain summaries, grouping similar domains together to reflect the four sections of this paper, and drafted the qualitative findings, including identifying supporting quotes. We presented preliminary qualitative results to the full research team, including our community researchers, to obtain their feedback and support data interpretation. We then incorporated their perspectives, based on their lived experiences and participation in the research, into the final report of the qualitative research findings. Quotes are presented in the text using pseudonyms chosen by 19 participants and by the research team for 2 participants who did not select a pseudonym.

## Results

Over the course of the pilot, 522 people completed the interest form to apply to the ABP program; 198 (37.9%) were eligible for the program and were entered into the Abundance Drawing. Of those entering the drawing, 151 (76.3%) were selected to participate in the program. Of the 151 program participants, 120 (79.5%) consented to participate in the evaluation research study.

Participants were considered part of the evaluation sample if they completed the T1 survey (*n* = 116); 93 completed the T2 survey, 106 completed T3, and 89 completed T4. All survey respondents identified as women (Table 1). The majority identified as Black only (79.3%), with smaller proportions identifying as Pasifika only (5.2%) or multiple races (15.6%). Participants ranged in age from 16 to 47 years, with a median age of 30 years. The majority identified as straight (85.3%). Regarding relationship status at T1, 39.7% of participants were single. Many survey respondents had never had children before the current pregnancy (37.9%), while 29.3% had given birth once, 14.7% had given birth twice, and 13.8% had given birth three or more times. At time of enrollment in the ABP program, the average gestational age in pregnancy was 20.2 weeks. The average time between enrollment in the program and completion of the T1 survey was approximately 22.5 days.

**Table 1:** Demographic characteristics of survey participants at T1 (*n* = 116)

Characteristic	*n* (%)
Race	
Black only	92 (79.3%)
Pasifika only	6 (5.2%)
Black and Pasifika	3 (2.6%)
Black, Pasifika & other race(s)	1 (0.9%)
Black & other race(s)	14 (12.1%)
Age	
Under 18	2 (1.7%)
18–24	23 (19.8%)
25–29	36 (31.0%)
30–34	30 (25.9%)
35–39	17 (14.7%)
40+	8 (6.9%)
Current Gender Identity	
Woman	116 (100.0%)
Current Sexual Orientation	
Straight	99 (85.3%)
Bisexual	14 (12.1%)
Other	2 (1.7%)
Missing	1 (0.9%)
Relationship Status	
Not currently involved with or seeing anyone	46 (39.7%)
Dating	8 (6.9%)
In a serious relationship with someone	40 (34.5%)
Engaged	7 (6.0%)
Married	13 (11.2%)
Other	2 (1.7%)
Number of Previous Births	
0	44 (37.9%)
1	34 (29.3%)
2	17 (14.7%)
3 or more	16 (13.8%)
Missing	5 (4.3%)
Gestational Age (weeks)	
11–19	28 (24.1%)
20–26	61 (52.6%)
27–33	27 (23.3%)
Educational Attainment	
Some high school (9th to 11th grade)	12 (10.3%)
High school graduate, GED or equivalent	39 (33.6%)
Some college, junior or community college or vocational school	51 (44.0%)
College graduate (bachelors degree)	10 (8.6%)
Graduate degree (MA, MS, MBA, PhD, MD, JD, etc.)	4 (3.4%)
Student Status	
Not enrolled in school	93 (80.2%)
Full-time student	10 (8.6%)
Part-time student	13 (11.2%)
Employment Status	
Working for pay full-time (30 h or more per week)	40 (34.5%)
Working for pay part-time (Less than 30 h per week)	27 (23.3%)
Not working for pay or unemployed	49 (42.2%)
Household Size (adults & children)	
1	14 (12.1%)
2	27 (23.3%)
3	36 (31.0%)
4	20 (17.2%)
5 or more	16 (13.8%)
Missing	3 (2.6%)
Monthly Household Income	
$0	10 (8.6%)
$1- $999	10 (8.6%)
$1000-$1999	18 (15.5%)
$2000-$3999	34 (29.3%)
$4000-$5999	9 (7.8%)
$6000 or more	10 (8.6%)
Missing	25 (21.6%)
Current Health Insurance	
Medi-Cal, Medicaid, Medicare, or any other government-sponsored health plan	89 (76.7%)
Private health insurance	26 (22.4%)
Do not currently have health insurance	1 (0.9%)
Other Benefits Programs Used Within the Past 30 Days	
CalWORKS (Cash Aid)/General Assistance (GA)/TANF	39 (33.6%)
CalFresh/SNAP/Food Stamps	75 (64.7%)
WIC	57 (49.1%)
Other benefits	92 (79.3%)
County of Residence	
San Francisco	115 (99.1%)
Contra Costa	1 (0.9%)

In terms of education, 44.0% had some college or vocational training, and 33.6% had a high school diploma or equivalent. Most respondents were not enrolled in school at T1 (80.2%). Most respondents (83.6%) were living in a household of four or less, and nearly all (99.1%) resided in San Francisco. Monthly household income ranged widely, with the largest proportion (29.3%) earning between $2,000 and $3,999. Over half (57.8%) of respondents were employed full-time or part time. Most respondents (76.7%) were enrolled in Medi-Cal or other government-sponsored health plans. Many respondents utilized public assistance programs within the past 30 days of T1, including CalFresh/SNAP (64.7%) and WIC (the Special Supplemental Nutrition Program for Women, Infants, and Children; 49.1%).

Of the 116 ABP participants who took part in the evaluation research, 21 participated in qualitative interviews; 20 participants completed a prenatal interview, and 19 completed a postpartum interview. One interview participant who experienced a preterm birth was interviewed only during the postpartum period. Sixteen interview participants identified as Black, four identified as Pasifika, and one identified as both Black and Pasifika. Eight interview participants reported having unstable housing, and two reported food insecurity in the T1 survey. Six interview participants were not receiving any kind of public assistance other than the ABP supplement. Fifteen interview participants had been pregnant before.

### Experiences with application and enrollment

Survey respondents generally reported positive experiences with the program (Table 2). When asked at T1 if ABP staff provided clear information regarding the application and selection process, most respondents either strongly agreed (72.4%) or agreed (25.9%). Similarly, 74.1% strongly agreed and 24.1% agreed that they were informed about how the program might affect their eligibility for other benefits (T1). Gathering the necessary eligibility documents was also considered very easy or easy by 58.6% and 31.0% of respondents, respectively, while 6.0% found it difficult and 2.6% found it very difficult (T1). At T1, survey respondents overwhelmingly felt respected by the Abundance Coaches, with 95.7% reporting they were treated very respectfully. At T2, the majority of respondents reported that the overall application process was easy (37.6%) or very easy (58.1%) and that the verification process was appropriately stringent (92.5%).

**Table 2:** Participant satisfaction with ABP program application, eligibility, and program start at T1 and T2

	*n* (%)
*T1 (n = 116)*	
How much do you agree or disagree with the following statement? *The ABP program staff provided you with clear information about how the application and selection process works.*	
Strongly agree	84 (72.4%)
Agree	30 (25.9%)
Neither agree nor disagree	0 (0.0%)
Disagree	0 (0.0%)
Strongly disagree	1 (0.9%)
Missing	1 (0.9%)
How much do you agree or disagree with the following statement? *The ABP program staff provided you with clear information about how your eligibility for other benefits programs may be impacted.*	
Strongly agree	86 (74.1%)
Agree	28 (24.1%)
Neither agree nor disagree	0 (0.0%)
Disagree	0 (0.0%)
Strongly disagree	0 (0.0%)
Missing	2 (1.7%)
How easy or difficult was it for you to gather required eligibility documents for ABP, like your pregnancy verification letter?	
Very easy	68 (58.6%)
Easy	36 (31.0%)
Difficult	7 (6.0%)
Very difficult	3 (2.6%)
Missing	2 (1.7%)
Do you feel that you are treated respectfully by the ABP program staff?	
Yes, very respectfully	111 (95.7%)
Yes, somewhat respectfully	3 (2.6%)
No, not very respectfully	0 (0.0%)
No, not respectfully at all	0 (0.0%)
Missing	2 (1.7%)
*T2 (n = 93)*	
Overall, how easy or difficult was it for you to go through the application process for the ABP program?	
Very easy	54 (58.1%)
Easy	35 (37.6%)
Difficult	3 (3.2%)
Very difficult	0 (0.0%)
Missing	1 (1.1%%)
Did you think the verification of eligibility process for the ABP program was too strict, not strict enough, or just right?	
Too stringent	5 (5.4%)
Not stringent enough	1 (1.1%)
Just right	86 (92.5%)
Missing	1 (1.1%)

Findings from the qualitative interviews support and lend context to the survey results. Most interview participants described the ABP application process as easy. For example, one participant, Cree, said, *“So applying for the program is easy. It’s very simple. It’s not difficult. [It’s] not a math*,* science situation. It’s just straightforward. And I love that.”* Another participant, MJ, noted that the application process was easy due to how clearly the Abundance Coaches described it to her: *“And then everything’s laid out very easily*,* like a checklist. I’m not reading a novel on what I need to provide…Everything’s very cut [and] dry.”* When discussing enrollment, a few participants cited the “no-strings-attached” nature of the program, including the lack of restrictions placed on how funds could be used and the limited amount of personal information needed to enroll (e.g., no social security number required), as a positive aspect of the enrollment process compared to other programs. As Brittany mentioned, *“It wasn’t a lot of questions asked. Like [other programs]*,* that they’re all in your business. They want to know how many breaths of air you’re taking every five seconds*.” Another participant noted that with ABP, she didn’t have to jump through the various hurdles that are characteristic of public benefits programs:

Getting into ABP was quick. With the other programs, you had to go through all these processes and [wait] to hear back from someone. If you don’t, you have to call and keep checking in. And then you gotta wait, and maybe they’re going to get back, maybe not. It’s just a lot… You have the recertification. Then you have all these check-ins. You get paperwork in the mail that don’t make sense. And when you call, you’ve got to keep waiting on hold. There’s five people in front of you, and nobody can help you at this time. Leave a name and number, we’ll get back to you, and then they don’t get back to you.

-Maya.

When asked what worked well about ABP, Maya went on to say: *“I feel like anytime I reach out to them through email or anything I feel like I get a response back*,* that I don’t have to wait for a long time to hear back from somebody.”*

Conversely, a quarter of interview participants experienced challenges during the enrollment process, including technical issues with the online dashboard used for program application and status tracking, the need to follow up with Abundance Coaches when no response was received, and the collection of all required documents for eligibility verification. Some of these participants noted that acquiring the required pregnancy verification documentation was particularly challenging, while others faced difficulties obtaining documents for proof of residence. After learning more about the onboarding process, Victoria said, *“It wasn’t as easy as pie as the girl [at a recruitment event] made it seem*,* you know*,* just apply*,* and you get it.*” Notably, two participants who reported having unstable housing at the time of applying, and thus were particularly overwhelmed, expressed difficulty with the eligibility verification process, suggesting that these participants may have experienced unique barriers to enrollment.

Interviewees who used other public benefits programs did not express concern about becoming ineligible for those programs due to enrolling in ABP. This was due to the benefits counseling provided by the Abundance Coaches, during which they informed participants of potential impacts on their eligibility for other benefits resulting from receiving the income supplementation. This was also due to the waivers the ABP program obtained from most public benefits programs, which ensured that participants’ eligibility and/or enrollment was not impacted by their enrollment in ABP. For example, when asked if she was worried about losing any other benefits she was using because of receiving the ABP income supplement, Tee Tee said: “*No. Because we’ve already went through the things that they cannot take away from you for receiving your [ABP supplement]*,* like with CalWORKs and all that stuff. So no*.” This participant described how her Abundance Coach provided benefits counseling and explained the potential implications of enrolling in ABP, which alleviated her concerns about losing her existing benefits by enrolling in the program.

### Experiences with Abundance Coaches

Most (95.7%) survey respondents reported being very satisfied with the service provided by the Abundance Coaches (T2; Table 3). When asked at T4 about the regular ABP program check-ins (which occurred two weeks after enrollment, during the 3rd trimester, and 1 month after delivery), the majority of survey respondents (83.1%) indicated that these meetings were very helpful. In addition, the majority of survey respondents had been connected to useful resources (e.g., Black Infant Health, Homeless Prenatal Program, doula services) by Abundance Coaches during their pregnancy at T3 (82.1%) and T4 (66.3%). Almost half of respondents (48.3%) had received Abundance Coaching at T4. For those who received Abundance Coaching, nearly all (90.7%) expressed interest in continuing it after the program ended, with ongoing support in the form of check-ins, financial advice, and connection to resources being the most desired forms of assistance (T4).

**Table 3:** Participant satisfaction and trust during program, A bundance Coaching, and program completion at T2, T3, and T4

	*n* (%)
*T2 (n = 93)*	
How comfortable do you feel receiving the ABP monthly income supplement?	
Very comfortable	79 (84.9%)
Somewhat comfortable	12 (12.9%)
Neither comfortable nor uncomfortable	1 (1.1%)
Somewhat uncomfortable	0 (0.0%)
Very uncomfortable	0 (0.0%)
Missing	1 (1.1%)
How much do you agree or disagree with the following statement? *There are no strings attached to receiving the $1000 income supplement every month.*	
Strongly agree	46 (49.5%)
Agree	38 (40.9%)
Neither agree nor disagree	5 (5.4%)
Disagree	2 (2.2%)
Strongly disagree	2 (2.2%)
How satisfied are you with the services provided to you by the Abundance Care Team?	
Very satisfied	89 (95.7%)
Somewhat satisfied	3 (3.2%)
Neither satisfied nor unsatisfied	0 (0.0%)
Somewhat unsatisfied	0 (0.0%)
Very unsatisfied	1 (1.1%)
*T3 (n = 106)*	
Have you been connected to any useful services by the ABP Abundance Care Team?	
Yes	87 (82.1%)
No	18 (17.0%)
Missing	1 (0.9%)
*T4 (n = 89)*	
How helpful did you find the ABP program check-ins, like the third trimester and postpartum check-ins?	
Very helpful	74 (83.1%)
Somewhat helpful	10 (11.2%)
Somewhat unhelpful	1 (1.1%)
Very unhelpful	3 (3.4%)
I did not have these check-ins	1 (1.1%)
Since you gave birth, have you been connected to any useful services by the ABP Abundance Care Team?	
Yes	59 (66.3%)
No	29 (32.6%)
Missing	1 (1.1%)
How much do you agree or disagree with the following statement? *I feel prepared for the ABP monthly stipends to end.*	
Strongly disagree	10 (11.2%)
Disagree	27 (30.3%)
Agree	38 (42.7%)
Strongly agree	14 (15.7%)
How do you feel about the amount of support you received from the Abundance Care Team specifically around the ABP monthly stipends ending? Was the amount of support you received…	
Too much	3 (3.4%)
Just right	76 (85.4%)
Not enough	7 (7.9%)
Missing	3 (3.4%)
How much can you trust the *ABP program* to make decisions that are in your best interest?	
A lot	69 (77.5%)
Some	17 (19.1%)
Only a little	2 (2.2%)
Not at all	0 (0.0%)
Missing	1 (1.1%)
How much can you trust the *government* to make decisions that are in your best interest?	
A lot	8 (9.0%)
Some	19 (21.3%)
Only a little	32 (36.0%)
Not at all	28 (31.5%)
Missing	2 (2.2%)
Did you receive Abundance Coaching from the Abundance Care Team?	
Yes	43 (48.3%)
No	42 (47.2%)
Missing	4 (4.5%)
*Received Abundance Coaching at T4 (n = 43)*	
Would you be interested in continuing Abundance Coaching even after the ABP program ends?	
Yes	39 (90.7%)
No	2 (4.7%)
Missing	2 (4.7%)
* Interested in continuing Abundance Coaching at T4 (n = 39)* What type of support would you like to continue receiving from the Abundance Coaching?	
Check-ins	12 (30.8%)
Financial support (coaching, credit building)	10 (25.6%)
Connection to other resources	9 (23.1%)
Mental health resources	2 (5.1%)
Any support	4 (10.3%)

Interview participants expressed appreciation for the genuine connection they felt with Abundance Coaches, noting that they were exceptionally kind and supportive, which helped alleviate stress during pregnancy and the postpartum period. For example, MJ reported that she was able to “vent” to the Abundance Coaches when she found out she could not have a home birth, and they encouraged her to use the ABP supplement for things that would help her relax, such as a spa day or a manicure. Other participants shared that they appreciated regular outreach from and check-ins with Abundance Coaches to ensure they had everything they needed from the program and that all of their program-related questions were answered. This was a positive contrast to some participants’ experiences with other programs; Kathy described interactions with Abundance Coaches as being “*just more friendly and more open and down to earth*,* how we are*,* you know*,* just being ourselves.”*

Interviewees noted that Abundance Coaches went above and beyond to provide them with holistic support, connecting them to outside resources that the program did not offer, such as housing services. One participant described this extra support, saying:

[The Abundance Coach] helped a lot. And just him helping me with my situation, as far as just being homeless and not having to worry about where I’m resting my head the next night, that helped a lot. It was easy to trust the program from there.

-Anonymous.

This interview participant described feeling like she could contact her Abundance Coach any time to help her address her immediate needs, and that they would be responsive to her. Interview participants described how Abundance Coaches supported them in developing a birth plan, applying for housing, and making connections to other programs, such as doula services, WIC, and breastfeeding and nutrition education. Other participants appreciated receiving gifts from the program, such as items for self-care and their new babies, as well as free family photo sessions offered to celebrate their pregnancy and postpartum journeys. Importantly, one participant reported that her Abundance Coach helped her navigate through her prenatal depression by checking in with her frequently, communicating with her beyond the program’s routine check-ins. Another participant described receiving support tailored to her as a Black mama, saying:

But man, it feels so good to be supported. [ABP has] done a lot to make sure that we’re cared about in a non-invasive way. I’ve felt a lot more connected to my experience as a Black mom. The fact that there is this program is really cool, because I think it’s not celebrated enough in our community, pregnancy.

-Victoria.

Notably, two interview participants did not receive support from ABP beyond the GI disbursement. One of these interview participants reported communication issues with the ABP program, while another said she did not feel she needed support, but knew she could get it from the Abundance Coaches, if an issue arose. Overall, interview participants felt supported by the Abundance Coaches, and cited a markedly different and positive experience compared to other benefits programs.

### Comfort with and trust in the program

During the program, participants reported high levels of comfort and satisfaction with ABP. A majority (84.9%) felt very comfortable receiving the monthly supplement. When asked how much they agreed with the statement that there were no conditions attached to the $1000 monthly supplement, 49.5% strongly agreed, and 40.9% agreed (Table 3). Participants were also asked: “How much do you trust *ABP* to make decisions in your best interest?” and “How much do you trust the *government* to make decisions in your best interest?” The majority of participants responded that they could trust ABP a lot (77.5%), whereas only 9.0% said the same about the government.

Many interview participants felt like they could trust the ABP program from the beginning, but some described being initially doubtful that the supplement would be “no strings attached.” While both the survey and interview data suggest that the overwhelming majority of participants trusted in the program and its Abundance Coaches, this sense of trust did not extend to the income supplement being unconditional. Notably, some attributed their skepticism to previous experiences with other benefits programs, in which participants had to provide excessive personal information or had to complete regular check-ins with the programs. For example, even though ABP was consistently described as no-strings attached, some still initially felt that they would eventually be expected to do something to receive the money, that they would have to repay it like a loan, or that their spending would be regulated in some way that was not apparent at the outset. Ariel noted, “*I thought it was going to be like everything else when they say*,* ‘Oh*,* no strings attached*,* blah*,* blah. Oh*,* but now you have to do this because if you don’t*,* we’re going to cut you off*.’” Similarly, Carla mentioned: *“No*,* I didn’t think [it would be no-strings attached]. I just said*,* it’s something. Something else has to be on the back end of this.”* Despite initial skepticism that the unconditional supplement was too good to be true given their past experiences, participants felt reassured once they actually received the funds on their debit card. When asked if she felt the GI supplement from ABP was really no strings attached, one participant said:

No, that I didn’t believe. At first, I was like, no. It’s got to be something to it, but as it’s been going on, I definitely think that there are no strings attached. And, I think that helps [to trust in the program] because, like I said previously, like with receiving childcare benefits and CalWORKs with my older daughter, there were strings attached.

-Britney.

Other participants noted that it was easy to trust the program because they saw themselves reflected in the Abundance Coaches and the program’s mission. When talking about what she liked about the program, one participant said:

They are more there to support me as a Black woman. And that’s a focus that they focus on. And it’s really important to me because it’s hard to find places and programs that do things like that and can focus on the needs of me as a woman of color. Because sometimes, I feel like I just get like pushed to the side.

-Angel.

Maya described why she felt she could trust the ABP program even before being selected into it, simply saying: *“It was people my color. I’m able to trust them.*” Lastly, a few interview participants indicated that they initially trusted ABP because they trusted the person who referred them to the program. As Britney described, *“I had a friend who had went through the program who actually referred me. So*,* from her experience*,* I trusted it.*” When friends, doulas, or other trusted service providers recommended ABP, these interviewees felt that they could trust the people behind the program, even if they still were skeptical that the income supplement would be unconditional, which speaks to the importance of community connections and word of mouth.

For many interview participants, the Abundance Coaches were key to facilitating trust in the program. For example, some described Abundance Coaches as having good intentions, genuinely wanting to help, and were communicative and responsive. As Melissa said, *“They seemed like pretty good people*,* like they would actually get back to me on time and not miss my calls or play phone tag. It was [easy] to get in contact with them.”* Generally, ABP participants appreciated the care demonstrated by the Abundance Coaches’ actions, which helped build a strong sense of trust. Specifically, they reported that the reliability and responsiveness of the Abundance Coaches, including responding to participants in a timely manner and following through on their commitments, were crucial in establishing this trust.

### Experience of program completion

As their time in the program came to an end, participants expressed concerns about their preparedness for the program to conclude. At T4, although most participants (85.4%) felt that the support they received regarding the end of the program was sufficient, only 42.7% reported feeling prepared for the GI supplements to end (Table 3). Interview participants were asked about their plans and preparation for the end of the supplements during both prenatal and postpartum interviews. During the prenatal interviews, many interviewees were planning to or had already started saving to soften the transition to no longer receiving the supplement. For example, Victoria said, *“I’m saving it for now so that when I don’t have it*,* I do have some cushion*,*”* while Britney said, *“I have set aside money knowing that it will eventually stop.”* These plans to save money felt within reach for some interview participants in the prenatal period, when participants had at least six more months of income supplements left, particularly for some who described how the additional income from the ABP program motivated them to budget their income and save as much of the income supplement as possible: *“[ABP] has helped me not only having money*,* but it helped me learn how to save*,* like literally. This has helped me a lot to learn on how to budget*,* ‘OK*,* I don’t need that*,* I don’t need that.’ That’s something I’ve always had a problem with.”*

-Kathy.

Many participants had stopped working in preparation for the birth of their new baby when they participated in prenatal interviews. Some participants mentioned that they planned to focus on finding a job to replace the additional income the supplement provided.

Once the baby gets here, I still have six more months of getting this income. So, I’ll make sure in that six months that I find a job so that I’m not worried about not getting that, [so that] I’m still going to have an income once those benefits stop.

-Anonymous.

To improve financial stability, some planned to participate in training or coursework to acquire new skills that would enable them to secure a better job or launch a career in a new field, thereby increasing their income. For example, when asked what she needed to reach her financial and career goals, Inga said:

I need to have a bachelor’s degree… I have to go to school basically, either way it goes. Anything I want to do next, I have to go back for my bachelor’s. So that’s, you know, a way to get both of those [jobs] going- is going back to school.

Participants were asked about their plans for the end of the program again during their postpartum interviews, when their income supplementation was nearly complete or had already ended. Many interview participants reported that they were seeking work or better paying employment to compensate for the end of their program participation. Some interview participants felt financially strained as they transitioned off of the program. Inga described a desire to change jobs to improve her financial situation, saying, “*My finances have changed dramatically almost. So*,* now I have to pick up where ABP left off. And I need to make something close to that with a job or something consistent*.” She later went on to describe the various expenses she had, which included baby food, formula and childcare: “*Everything is expensive. So*,* it’s just like*,* OK. I’m definitely hurting by not having [the supplement]*.” Some participants noted that they were simply returning to budgeting and managing their finances in the same way that had worked for them before entering the ABP program. Other participants felt more prepared for the program to to end due to the money management skills they had acquired through program participation, either informally from an Abundance Coach with previous experience working in financial literacy, or through referrals to outside organizations providing financial wellness services.

Overall, interview participants reflected that the GI supplements helped ease their stress and worry during pregnancy and postpartum, but it did not necessarily change their long-term financial situation. Interviewees found themselves in a similar place when the supplementation ended as when it began, but they were thankful for not having to stress about finances during the crucial time period of pregnancy and postpartum. Despite this, many described ABP as a “stepping stone” for them, laying the groundwork for future financial stability, either by helping them catch up on bills or by supporting them in developing financial management skills or reaching their goals:

*[ABP] is like a stepping stone. It’s like part of the ladder*,* and transitioning seems like it’s moving on to whatever’s going to be next. There’s going to be a next phase in my life. So*,* it was definitely a stepping stool. So my plans are just to continue to work towards my [career and educational] goals that the Abundant Birth Project has helped me to also feel closer to.*

-Victoria.

## Discussion

The ABP San Francisco pilot was the first GI program in the U.S. designed specifically to address economic insecurity during pregnancy, with the goal of disrupting birth inequities. These findings indicate that participants overwhelmingly found the program to be acceptable. Participants highly valued the ABP program, demonstrating strong trust in the Abundance Coaches and reporting high satisfaction with the application and enrollment process, and support services provided through the program. Though participants initially harbored skepticism about the GI supplement being truly unconditional and unrestricted, rooted in past experiences with public assistance programs that often had extensive requirements to fulfill and that closely monitored how recipients used their benefits, their positive experiences with the program ultimately overcame this skepticism.

This study adds to the limited literature on the acceptability of GI programs. While other programs in the most current wave of GI pilots have examined the impact of their programs on health and financial outcomes, few have reported on participants’ experiences navigating GI programs. David et al. (2024) explored participants’ experiences with the Ontario Basic Income Pilot, which launched in 2017. Participants in the program reported that they felt it was more generous than Canada’s public assistance programs. The minimal behavioral conditions and reduced monitoring allowed them to feel trusted by the state, in stark contrast to their previous experiences with Canada’s dehumanizing public assistance systems [21]. Our findings mirror this, as ABP participants felt that they could trust and were trusted by the program and that the unconditional nature of ABP was refreshingly different from public assistance programs they had used in the past.

In 2022, in response to the growing movement in support of guaranteed income, the California Department of Social Services (CDSS) funded GI programs across the state to test whether unconditional cash payments, as a supplement to other benefits, could positively impact participant well-being, particularly among pregnant people and foster youth [22]. The California expansion of ABP was one of seven programs selected to participate in this statewide funding initiative, expanding the program to Los Angeles, Riverside, Alameda, and Contra Costa Counties starting in January 2024. Evaluation of the California ABP expansion is currently underway. Early implementation findings about participant experiences with the CDSS-funded programs indicate that program participants who had applied and started receiving the GI payments described the application process as easy and fast, with some pregnant participants citing challenges with getting proof of pregnancy [22]. This finding aligns with the current study’s results, where participants described the San Francisco ABP pilot’s application as easy to complete, although a few encountered issues with providing the required documentation.

Our findings also align with prior research documenting the challenges of accessing public assistance. Participants described past experiences with safety net programs as burdensome, stigmatizing, and bureaucratic—often requiring them to “jump through hoops” and sacrifice privacy for limited support, echoing findings from the literature on experiences with public assistance programs [23–28]. These past experiences shaped participants’ expectations of what was possible, leading many to question the unconditional nature of ABP not only during their time in the program but even during its design phase. To inform the design of ABP, needs assessment interviews were conducted with Black and Pasifika women who had experienced pregnancy in San Francisco [18]. These participants similarly described negative past experiences accessing public assistance, and also struggled to imagine a cash assistance program that was unconditional. Some expressed concern that recipients of such support might not use the funds in ways society deems “appropriate.” This skepticism reflects a deeply embedded, false, and harmful narrative: that public assistance recipients are inherently untrustworthy and must be monitored in exchange for aid [29–31].

Findings from the current study demonstrate that even among those who ultimately enrolled in ABP, skepticism about the program’s unconditional nature was also common at the outset, based on previous experiences with and understanding of public assistance programs. Many participants struggled to believe that a “no-strings-attached” income supplement was possible, given their prior experiences with restrictive and dehumanizing public assistance programs. These findings were similar to that found among participants of the CDSS-funded GI programs, who also expressed initial skepticism about the program, saying it seemed “too good to be true” or a “scam”; this skepticism faded once trusted people—Abundance Coaches, social workers, and friends who were familiar with the programs—assured them the program was indeed legitimate [22]. Indeed, with the San Francisco ABP pilot, program participants’ perceptions shifted over time. As they experienced the program’s consistency, respectfulness, and lack of punitive oversight, ABP participants’ trust in the program grew.

These findings support the theory that GI can help reduce the stigma associated with cash assistance [32–36]. This topic remains understudied in current GI pilots across the U.S., making these results an important contribution to the literature. The features of GI–unconditional and unrestricted payments–are counter to traditional public assistance programs, which often surveil and scrutinize recipients. In addition, pregnancy can be a time for increased surveillance, specifically for Black birthing people, who often have to endure being treated with suspicion and judgment by medical and social service systems [37, 38]. As a result, GI recipients have experienced a new model of support, and for ABP participants, a new model of perinatal support. ABP participants initially believed that cash assistance programs were conditional, but their experience with a GI program helped to reframe their understanding of what is possible. Furthermore, ABP was designed with and administered by members of the community, which built trust among participants [18]. The shift in participants’ mindsets demonstrates not only the transformative power of GI in changing perceptions of cash assistance but also highlights the importance of thoughtful, community-based program design and delivery in helping participants reimagine what supportive and dignified assistance can be.

Participants’ trust in ABP was deeply rooted in the program’s community-centered approach. Trust was not only built through positive interactions with Abundance Coaches, who were described as responsive, respectful, and reflective of the communities served, but also through the broader sense of alignment between the program’s mission and participants’ lived experiences as Black and Brown women. Many participants noted that “seeing themselves” represented in the Abundance Coaches and being referred by trusted community members enhanced their comfort and willingness to engage with the program, even when initial skepticism remained about the unconditional nature of the income supplement. The factors behind participant trust in the ABP program mirror research on other community-centered programs. In particular, Community Health Worker (CHW) and Promotores models share a similar approach to fostering trust among participants. For example, Vanden Bossche et al. (2022) found that trust between CHWs and vulnerable clients was fostered through mechanisms of recognition, equality, and reciprocity, similar to participant experiences in ABP [39]. Citing the importance of feeling seen and understood by Abundance Coaches who reflected their identities and values, ABP participants described interactions as humanizing rather than bureaucratic or transactional. In both models, trust was fostered through intentional design rooted in community needs.

The San Francisco ABP program was a small-scale pilot that enabled agility and adaptation to align with community needs in real-time. For instance, when the program first began, some early participants reported difficulty in gathering the necessary eligibility documents, and adjustments to the program were made in real-time based on that feedback. A future challenge may be in transitioning the program to a larger scale. Indeed, prior research has cited the “tradeoffs” that often need to be made when scaling up smaller health interventions, sometimes at the expense of program fidelity [40, 41]. For example, Abundance Coaches provided personalized and responsive support tailored to each participant’s needs. On a larger scale, this level of individualized support may not be feasible due to budgetary constraints that could hinder face-to-face time between coaches and clients. Additionally, ABP’s recruitment and coaching strategies were intentionally designed to meet the needs of Black and Pasifika pregnant people in San Francisco. As the program expanded to new counties, these approaches were adapted to reflect the cultural contexts and priorities of the specific communities being served. Community-centered work requires time, trust-building, and adequate funding, which may be difficult to secure within limited timelines and budgets.

A limitation of ABP, but also of any GI pilot program, is the inability to create lasting change through continued financial support. Some participants described feeling supported by the Abundance Coaches throughout the program’s graduation process, but still experienced distress because the payments were coming to an end. Abundance Coaches provided guidance and connections to resources as the program’s conclusion approached, but, as a time-limited pilot program, ABP could not necessarily offer lasting change for participants and recognized that systems changes were needed to address the ongoing economic insecurity that the study participants were navigating. This highlights a fundamental challenge also recognized by other GI pilot programs; while they can offer short-term support during a critical window, sustainable economic solutions require policy-level systemic changes [42–45].

### Strengths and limitations

The ABP program and the evaluation were co-created with Black and Pasifika communities, a notable strength that ensured both were responsive to the needs of each community from start to finish. Community researchers played a crucial role in instrument development, data collection, and analysis, which helped foster trust among evaluation participants. In addition, the mixed-methods longitudinal approach, which included quantitative surveys and qualitative interviews administered throughout the pregnancy and postpartum periods, provided a holistic understanding of participants’ experiences with the program over time. The study also had some weaknesses. Social desirability and recall bias may have influenced responses, as all data were self-reported. There may also be potential selection bias, given that the evaluation was opt-in. Consequently, participants in the evaluation may differ from the rest of the ABP program participants in that they may have felt more comfortable and inclined to share their experiences, whether positive or negative. Lastly, lower response rates in the later surveys may be a limitation, since the findings reflect the experiences of those who stayed engaged over time, rather than the full sample. Despite these limitations, the study’s findings provide evidence of the acceptability of a community-centered perinatal GI program.

### Future research directions

Several unanswered questions from this study could be explored in future research. Although this study provided evidence that participation in ABP may have shifted recipients’ views of cash assistance, it remains unclear whether these changes in perceptions are long-term. Future longitudinal studies should explore whether shifts in perceptions of cash assistance remain among GI pilot recipients after income supplementation ends. Furthermore, it is unclear whether the change in perceptions resulted from ABP’s community-centered design and administration, the income supplement itself, or a combination of both. Additional studies should explore whether the changes seen in perceptions among ABP participants were unique to community-centered GI pilots or can be generalized to all GI pilot models.

Lastly, with the expansion of ABP, there have been concessions made that have limited the fidelity to the original program design. Future research on the expansion of the program should explore the changes that were necessary due to the constraints of public funding and how those changes may have impacted service delivery to participants.

## Conclusions

ABP was the first perinatal GI pilot program in the U.S. and was designed with and for Black and Pasifika communities in San Francisco. Program participants trusted the program due to its community-centered approach and genuine care for them, in stark contrast to the distrust they held for many government assistance programs. The program’s emphasis on dignity, responsiveness, and cultural alignment not only helped participants feel seen and supported during pregnancy and childbirth but also allowed participants to experience a program that challenges dominant narratives about who deserves support and under what conditions. While skepticism about the unconditional nature of the program was initially present, participants ultimately came to trust both the program and the cash supplement itself. These findings highlight the transformative potential of GI programs that are rooted in community and implemented with respect and intentionality. As GI pilots expand across the country, ABP provides an important model for designing and delivering support that centers the voices and needs of those most impacted by health inequities.

## Data Availability

The datasets generated and/or analyzed during the current study are not publicly available due to the sensitive nature of the data and the strong risk of deductive disclosures given the small and specific population of focus. The data may be available from the corresponding author upon reasonable request.

## Abbreviations

ABP: Abundant Birth Project
GI: guaranteed income
TANF: Temporary Assistance for Needy Families
SNAP: Supplemental Nutrition Assistance Program
WIC: Special Supplemental Nutrition Program for Women, Infants, and Children
CDSS: California Department of Social Services
